# Comparing the traditional and Multiple Mini Interviews in the selection of post-graduate medical trainees

**Published:** 2015-12-11

**Authors:** Michael C Sklar, Antoine Eskander, Kelly Dore, Ian J Witterick

**Affiliations:** 1 Department of Anesthesiology, University of Toronto; 2 Department of Otolaryngology – Head & Neck Surgery, University of Toronto; 3 Deparment of Biostatistics, McMaster University

**Keywords:** Multiple mini interview, medical education, traditional interview, postgraduate admissions

## Abstract

**Background:**

The traditional, panel style interview and the multiple mini interview (MMI) are two options to use in the selection of medical trainees with each interview format having inherent advantages and disadvantages. Our aim was to compare the traditional and MMI on the same cohort of postgraduate applicants to the Department of Otolaryngology – Head & Neck Surgery at the University of Toronto.

**Method:**

Twenty-seven applicants from the 2010 Canadian Residency Matching Service selected for interview at the University of Toronto, Department of Otolaryngology – Head & Neck Surgery were included in the study. Each applicant participated in both a traditional interview and MMI.

**Results:**

Traditional interviews marked out of a total maximum score of 570. On the traditional interview, scores ranged from 397–543.5 (69.6 – 95.3%), the mean was 460.2. The MMI maximum score was out of 180. MMI scores ranged from 93 – 146 (51.7 – 81.1%) with a mean of 114.8. Traditional interview total scores were plotted against MMI total scores. Scores correlated reasonably well, Pearson Correlation = 0.315 and is statistically significant at p = 0.001. Inter-interview reliability for the two interview methods was 0.038, with poor overall agreement 0.07%.

**Conclusions:**

MMI and traditional interview scores are correlated but do not reliably lead to the same rank order. We have demonstrated that these two interview formats measure different characteristics. One format may also be less reliable leading to greater variation in final rank. Further validation research is certainly required.

## Introduction

A growing body of knowledge is attempting to describe and substantiate the optimal means for the selection of candidates into a medical training program. Much of the selection of medical trainees, particularly at the post-graduate level, depends on the selection committee’s assessment of that applicant’s attributes both so-called cognitive and non-cognitive.* The traditional panel style interview that has long been the mainstay of assessing inter-personal and behavioural attributes and characteristics has been scrutinized with limitations cited due to interviewer bias,^1^ the lack of psychometric robustness and questionable reliability and validity.^2^ As a result, selection panels have sought a more optimal platform for the assessment of applicants’ non-cognitive traits. The advent and first description of an alternative medical admissions interview, the Multiple Mini-Interview (MMI), comes from work done at McMaster University.^3^

Cognitive attributes have traditionally been assessed through performance on written tests and grade point average while measures of other attributes have been assessed by means of letters of reference and traditional interviews.^4^ It is often difficult to assess cognitive attributes during medical school as most Canadian medical schools have adopted pass/fail or honors/pass/fail systems. Until recently, medical educators largely relied on a panel-style interview to assess the inter-personal and behavioural attributes and characteristics of an applicant. Since its inception in 2004, there has been mounting evidence supporting the use of the MMI in medical school admissions in place of the TI. Early feasibility data from the MMI demonstrated a reliability of 0.65 on a cohort of undergraduate medical applicants and that this statistic was consistent with other admissions criteria.^3^ Evidence suggests that the MMI is a superior assessment tool as compared to the TI because of its ability to hone in on specific skills and attributes. Furthermore, it is regarded favorably by both applicants and interviewers with significant potential for cost saving.^3^,^5^,^6^ The MMI is also associated with performance on the Canadian licensing exam (Medical Council of Canada Qualifying Examinations^7^ - MCCQE) which supports its ability to predict both cognitive (MCCQE step 1) and non-cognitive traits (MCCQE step 2) necessary for patient care.

Undergraduate and postgraduate admissions processes are similar but differ significantly in applicant pool size and structure. Postgraduate pools are smaller and more homogeneous.^8^ Currently, there are only three studies in the medical literature which describe the use of the MMI in the selection of postgraduate trainees. The first study, from the University of Calgary, describes a cohort of international medical graduates (IMGs) who participated in an MMI for entrance into Family Medicine postgraduate training. This study revealed a moderate correlation but could not demonstrate a correlation between interview scores and exam performance, which argues against the MMI’s predictive value.^9^ The second study assessed the reliability of the MMI in Canadian medical graduates and IMGs for entrance into residency training in Obstetrics and Gynecology, Pediatrics and Internal Medicine at McMaster University and the University of Alberta.^8^ The overall reliability (kappa) of the MMI ranged from 0.55–0.72, which indicates moderate reliability. The authors conducted surveys as well, in which 88% of candidates believed they could accurately portray themselves during the MMI, and 77% indicated that specialized medical knowledge was not needed to complete the stations. Finally, 74% believed the MMI outperformed the TI method. The most recent study^10^ assessed the reliability and feasibility of the MMI for selection of postgraduate trainees in Physical Medicine and Rehabilitation at the University of British Columbia and demonstrated sufficient inter-rater reliability in three out of their four MMI stations while the overall MMI interview had moderate reliability. The unifying limitation of these studies is the lack of a true control group as none of these applicants underwent a TI simultaneously with their MMI.

There have been only two studies, both for MD program admissions, examining performance on the MMI and TI by the same cohort.^11^,^12^ A study from McGill University simultaneously administered a TI and MMI. The MMI was rated more highly by applicants on fairness, imposition of stress and effectiveness as a measurement tool.^12^ This study did not however compare scores for these two interview techniques. O’Brien *et al.*^11^ ran TIs alongside MMIs in the United Kingdom. Their applicant pool was comprised of two different undergraduate streams, a five-year and a four-year program. The authors found that scores for their four-year program cohort did not significantly differ for the MMI or TI. The five-year cohort however, performed better on the MMI. Intra-class correlation coefficients for the two cohorts were both significant at 0.69 and 0.73 respectively. Finally, the authors found mixed results from the two groups with respect to the interview evaluations, with the four-year group favoring the TI and the five-year group favoring the MMI. These two studies demonstrate that the MMI may be acceptable for use in undergraduate MD admissions, but they provide little evidence that it is superior to the TI.

Few studies have compared the MMI to the traditional interview (TI) in the selection of postgraduate medical trainees. To date, the majority of MMI literature comes from undergraduate medical school admissions research.^2^,^4^–^7^,^12^,^13^ The optimal assessment of attributes other than those embodied in grades and performance reviews of medical trainees applying for residency training is not yet known.

Our objective was to compare the MMI and TI in the same cohort of applicants applying for postgraduate training in the Department of Otolaryngology – Head & Neck Surgery at the University of Toronto. We aimed to objectively compare the two major modalities of non-cognitive assessment of medical trainees at the postgraduate level. We focused on whether these result in correlated interview scores and whether these lead to congruent rank lists.

## Methods

For the 2010 Canadian Residency Matching Service (CaRMS) postgraduate application process, the Department of Otolaryngology – Head & Neck Surgery at the University of Toronto introduced for the first time a dual interview system for the selection of postgraduate trainees. Ethics approval was obtained to develop and administer the dual interview procedure. All interviews were conducted on a single day and the order in which applicants participated in either the TI or MMI was randomly assigned.

The TI consisted of three stations that were each fifteen minutes in duration. There were two raters per traditional station; two stations had two faculty each and one station had two residents (PGY3 and 5). Traditional interviewers were provided with the application packages of the prospective students prior to the interview. Each traditional interviewer could award an applicant a maximum score of 95 points, thus making each traditional interview worth a maximum of 570 points. The resident station also included a 5 mark surgical skills task. For the 2010 cohort, students were tasked with completing a simple interrupted and horizontal mattress suture on a piece of synthetic skin. Questions in the traditional interview focused mainly on applicant background, interest and motivation to pursue a career in otolaryngology – head & neck surgery, extracurricular activities and research activities.

The objectives of the MMI stations were similar to those previously described; evaluation of communication and presentation skills, decision making, and the ability to think critically and to debate a complex issue (skills that clearly required higher level thinking and were not in any way “non-cognitive”).^11^ Assessors were also given an opportunity to raise a “red flag;”^2^ an opportunity to express severe concerns about a candidate’s suitability. Scenarios for the 2010 MMIs were based on the following themes: interprofessionalism, the ethical use of the internet, discussion of the CanMEDS competencies, managing an awkward situation, a controversial cancer drug, and preferential access to health care. The MMI portion of the interview consisted of six ten-minute stations, each with a single rater. MMI interviewers were blinded to the applicants’ backgrounds and other application material and had received only the name of the student they would be interviewing. The MMI scenarios were selected from a wide range of scenarios already in use at McMaster University for undergraduate medical education candidate selection. They represented a broad range of competencies that were selected for their relevance to traits the CaRMS selection committee felt desirable in otolaryngology – head & neck surgery candidates. Five of the stations were administered by faculty and one of the MMI stations was resident evaluated. Prior to the MMI, staff and resident evaluators participated in formal training sessions for MMI evaluators. Each MMI station was scored out of 30 possible points. Ten points were assigned to each of the following three components per interview station: communication skills, ability to construct an argument and overall performance. Thus, each candidate could score a maximum of 180 points on the MMI.

Residents and faculty were asked to provide feedback to the selection committee on any candidates they worked with on clinical rotations. This information was collected 1 to 3 weeks before the interviews and not provided to the committee until all TI and MMI scoring was complete. The final rank order was carried out by consensus by the six TI interviewers after reviewing all of the available data (TI scores, MMI scores, surgical skills score and resident and faculty comments about candidates). To assess whether the TI and MMI scores correlated and led to the same rank order, statistical analysis was carried out using SPSS Statistics Software, version 20 (IBM Version 20.0. Armonk, NY: IBM Corp). Median, range, mean, and standard deviation were calculated for each traditional interviewer, each MMI station and for the total overall MMI and TI scores. Total MMI and TI scores were also converted into percentages based on the maximum possible score for each applicant. Pearson’s correlation coefficient was calculated to describe the correlation between traditional and MMI scores. A *p*-value of less than 0.05 was used to define statistical significance. Each interview total score was used to rank applicants from highest to lowest for each interview technique separately. We then used kappa statistics to test the inter-interview reliability of the two interview techniques.

Candidates assessed their experience with the MMI and TI through confidential surveys. Both the MMI and TI survey consisted of seven questions and each question was graded on a seven-point Likert scale (Table 1).

## Results

For the first iteration of the 2010 CaRMS match for Otolaryngology – Head & Neck Surgery at the University of Toronto, the program received 40 applications. All applications were screened by a selection committee and 27 applicants were invited for interview. The applicants were informed of the interview format (TI and MMI) by letter and all 27 accepted the invitation for interview. This cohort of 27 applicants represented 12 Canadian undergraduate medical institutions: seven interviewees from one institution, three institutions with three interviewees each, three institutions with two interviewees each, and five institutions with a single interviewee.

Tables 2 and 3 illustrate candidate performance on the traditional interview and MMI respectively. Each of the six traditional interviewers gave a score out of 95 possible points to each applicant for a total maximum score of 570. On the traditional interview, total scores ranged from 397–543.5 (69.6 – 95.3%). The mean total traditional score was 460.2 with a standard deviation of 5.98. Similarly, each of the six MMI stations was marked out of 30 possible points for total maximum score of 180. On the MMI, total interview scores ranged from 93 – 146 (51.7 – 81.1%). The mean MMI score was 114.8 with a standard deviation of 12.5. There were no “red flags” raised on any of the 27 interviewees.

**Figure 1:**
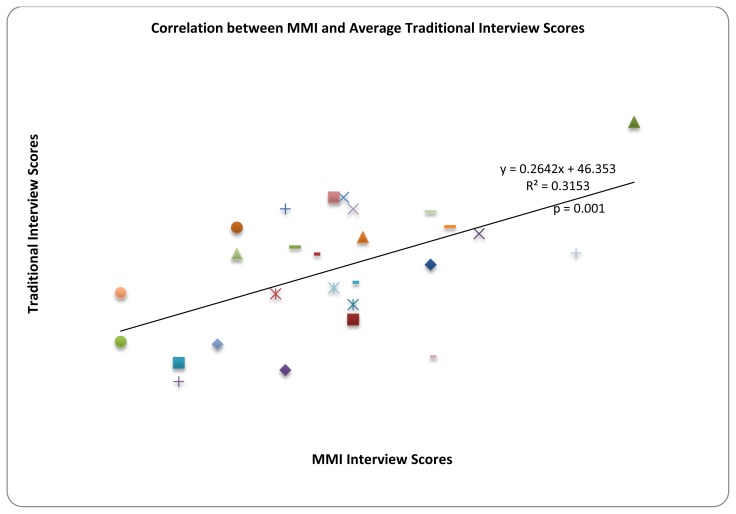
Traditional interview total scores plotted against MMI total scores. *R^2^* = 0.315, *p* = 0.001. Each symbol represents one candidate’s MMI score plotted against traditional interview score.

Average traditional total scores were plotted against total MMI scores for each candidate (Figure 1). A Pearson correlation coefficient calculation yielded a statistically significant moderate correlation (*r* = 0.315; *p* = 0.001).

Table 4 categorizes candidates by MMI rank in descending order and their respective rank on the traditional interview. Although the two scoring methods were moderately correlated (Figure 1) there was a very poor inter-interview agreement on final rank (Table 4) as demonstrated by a kappa statistic of 0.038. The interview survey responses were categorized according to morning and afternoon MMI and a single TI sitting (Table 1).

## Discussion

The selection of medical trainees both at the undergraduate and postgraduate levels can be challenging and is not an exact science. Educators experience great difficulty in selecting the “best” candidates from a relatively homogenous pool of highly qualified applicants. Ultimately, selection relies on the assessment of the so-called cognitive and non-cognitive attributes of the applicant.

Our study is the first to compare the MMI and TI in the same postgraduate applicant cohort. In doing so, we have observed that MMI and TI techniques are correlated in score but do not reliably lead to the same rank order. We have shown that these two interview techniques measure different characteristics as demonstrated by the variations in rank order. Alternatively, one technique may be less reliable leading to greater variation in final rank. Using factor analysis to correlate scores between their MMI stations, Lemay *et al.*^6^ showed that the attributes of advocacy, ambiguity, collegiality and collaboration, empathy, ethics, honesty and integrity, responsibility and reliability, and self-assessment could be independently evaluated in the MMI setting. We believe that many of these core qualities were evaluated in our MMI as well. In the TI, candidates were also given the opportunity to discuss their educational background, extracurricular activities and desire to be an otolaryngologist – head & neck surgeon.

Interviewee responses to the two interview types were generally similar with some important exceptions. All TI respondents felt that this type of interview allowed for accurate portrayal of their abilities, compared with a smaller proportion feeling this way about the MMI. In addition, most felt that the MMI was anxiety provoking as compared to the TI. As this is the first such dual interview process at our institution, it is difficult to determine whether these differences observed are due to inherent differences in the interview types, or whether this dichotomy exists secondary to the unfamiliarity and lack of experience with the MMI style interview.

The development of the final rank order list of the candidates warrants discussion. Candidates are evaluated by several mechanisms. The interview portion, as previously mentioned, consists of the TI, MMI and surgical skills station. The final ranking committee reviewed the interviewee application files: the details of electives, medical school transcripts, reference letters, curriculum vitae and letter of intent. Following the interviews, resident and faculty comments about the interviewed candidates were reviewed. All of this information was reviewed by the rank committee and the final rank order list was subsequently generated. Final candidate ranking is a combination of file reviews, interviews, comments and debate.

The present study has several limitations. Firstly, our sample size of 27 is small when compared to studies assessing undergraduate applicants. Our sample is from a single CaRMS cohort applying to a relatively small postgraduate training program. When compared to other otolaryngology - head & neck surgery programs in Canada, this would actually represent the largest applicant cohort in the country. Secondly, bias likely affects the final ranking of candidates. Traditional interviewers are not blinded to candidates, in that personal letters and applications are thoroughly reviewed prior to interviews. There is likely less bias with the MMI because these interviewers are only given the candidates’ names. Final rank order lists are generated through selection committee deliberation and analysis of discordant interview scores. These results are not included in our study as our goal was not to assess whether MMI or TI predicted rank but rather whether they were correlated and whether they led to the same rank order. Another weakness is the lack of objective results of how these applicants perform during residency through cognitive measures, for example through National In-Training Exams, and non-cognitive measure, for example, rotation evaluations. This, however, would be very difficult to remedy because only those with the highest MMI/TI scores gained entry to the program and thus we would lose data on the remaining 22 applicants in such an analysis.

Several questions also arise from the interview analysis that leads to further investigation. For instance, the specific MMI scenarios evaluate specific traits and skills, but the scenario is selected arbitrarily, without a test blueprint or overall plan. An evaluation of each specific scenario is warranted to determine if all MMIs are equal and what happens when different MMI scenarios are used to arrive at a final MMI score. Furthermore, what is the effect that previous experience with the MMI has on performance? Are those students who participated in an MMI for undergraduate medicine likely to do better on the postgraduate interview? In addition, we continue to speculate on how to combine the data from the two interview types if indeed candidates will be asked to participate in both. More research is needed to determine which information is important from each of the interview types and what selection committees are to do if there are discordant interview scores.

Despite the above limitations, this study adds to the medical education literature. Firstly, we have replicated a correlation between the MMI and TI previously reported in the literature. Secondly, this is the first study to test the two interview types on the same postgraduate applicant cohort. Finally, we offer caution to medical educators about the appropriateness of using one interview type over the other: the two may actually be measuring different attributes and synergistically provide more information to a selection committee than either interview alone.

## Figures and Tables

**Table 1:** Survey questions and answers for the MMI & TI

Survey Question	MMI	TI
Accurate portrayals of abilities	85%	100%
Anxiety provoking	63%	37%
Dual interview process as a deterrent to applications	0%	0%
Clear instructions	90%	100%
Required specialized knowledge to answer	56%	10%
Station difficulty	Neutral	Neutral
Appropriate time per station	64%	90%

**Table 2:** Median, range, mean and standard deviation scores for the six traditional interviewers, scored out of 95

Rater	1	2	3	4	5	6	Total
**Median (Range)**	75 (65.5–86.0)	78 (69.0–90.5)	68 (42.0–91.0)	75 (88.0–93.0)	83 (48.0–94.0)	82.5 (67.0–94.0)	469.2 (397–543.5)
**Mean (SD)**	75.5 (4.8)	77.2 (5.3)	68.5 (13.2)	74.9 (7.7)	81.9 (9.1)	82.2 (6.1)	460.2 (5.98)

**Table 3:** Median, range, mean and standard deviation scores for the six MMI scenarios, scored out of 30

	MMI 1	MMI 2	MMI 3	MMI 4	MMI 5	MMI 6
**Median (Range)**	22 (18–30)	20 (16–25)	15 (8–30)	22 (14–28)	17 (11–21)	17 (7–27)
**Mean (SD)**	22.2 (3.1)	20.7 (2.3)	16.0 (6.2)	21.7 (3.8)	17.1 (2.7)	17.0 (4.6)

**Table 4:** The rank order list

Candidate	MMI Rank	Traditional Rank
C	1	1
Y	2	12
D	3	9
R	4	7
A	5	15
Z	6	24
AA	7	6
L	8	10
B	9	21
E	10	20
Q	11	16
V	12	5
M	13	2
T	14	3
W	15	17
H	16	13
I	17	11
G	18	4
J	19	26
N	20	19
F	21	8
U	22	14
S	23	23
K	24	25
P	25	27
O	26	22
X	27	18

## Footnotes

* Editor’s note: The term “non-cognitive” is problematic. While the term has a long history in medical education and other disciplines and is widely used, it is clear that people do in fact think and rely on their prior learning when demonstrating the skills and attributes clustered under that awkward moniker. Furthermore, the dichotomy is overly simplistic, obscuring and conflating important distinctions and may inadvertently reinforce the prevalent belief that the “soft people skills” are not central to the work of being a doctor. Unfortunately there is no one term that seems to adequately describe what we have been lumping under “non-cognitive.” In this article, we have used a variety of terms in addition to non-cognitive. We hope a consensus will soon emerge.
